# Immunotherapy resistance: the answers lie ahead – not in front – of us

**DOI:** 10.1186/s40425-017-0212-y

**Published:** 2017-02-21

**Authors:** Miles C. Andrews, Jennifer A. Wargo

**Affiliations:** 1 Department of Surgical Oncology, The University of Texas MD Anderson Cancer Center, 1400 Pressler Street, Unit Number 1484, Houston, TX 77030 USA; 2 Department of Genomic Medicine, The University of Texas MD Anderson Cancer Center, 1400 Pressler Street, Unit Number 1484, Houston, TX 77030 USA

**Keywords:** Checkpoint blockade, Resistance, Longitudinal biopsies, Interferon, T cell exclusion

## Abstract

Mechanisms of innate and adaptive resistance to checkpoint blockade immunotherapy are under intense investigation with a view to broadening the therapeutic potential of this form of treatment. In a recent manuscript by Zaretsky and colleagues, mutational events were identified that effectively crippled ongoing immunotherapy responses in patients treated with anti-PD-1 therapy. These results are discussed in the light of other recent and ongoing research efforts exploring both mutational and non-mutational resistance mechanisms, highlighting the critical translational importance of longitudinal tumor sampling.

## Commentary

A commentary on: Zaretsky JM, Garcia-Diaz A, Shin DS, Escuin-Ordinas H, Hugo W, Hu-Lieskovan S, Torrejon DY, Abril-Rodriguez G, Sandoval S, Barthly L, Saco J, Homet Moreno B, Mezzadra R, Chmielowski B, Ruchalski K, Shintaku IP, Sanchez PJ, Puig-Saus C, Cherry G, Seja E, Kong X, Pang J, Berent-Maoz B, Comin-Anduix B, Graeber TG, Tumeh PC, Schumacher TN, Lo RS, Ribas A. “Mutations Associated with Acquired Resistance to PD-1 Blockade in Melanoma.“NEJM 2016;375(9):819–829.

Immunotherapy continues to gain traction as an effective therapeutic strategy across several cancer types. Much of the success has been demonstrated through the use of immune checkpoint blockade targeting cytotoxic T-lymphocyte associated protein 4 (CTLA-4) and programmed-death 1 (PD-1)/PD-1 ligand (PD-L1), with the highest objective response rates observed in cancer types with a high mutational burden such as melanoma and non-small cell lung cancer, likely related to an enriched neoantigen repertoire [1]. However significant limitations exist with these therapeutic agents when used as monotherapy, with objective responses to PD-1 blockade observed in only 30–40% of patients [2, 3], and the majority of patients demonstrating innate resistance. Acquired resistance to anti-PD-1 therapy is also a problem, with approximately one quarter of responders later demonstrating disease progression [4].

Significant efforts are underway to identify mechanisms of innate and acquired resistance to immune checkpoint inhibitors via translational research in human samples [5–7], and a recent study published in the New England Journal of Medicine by Zaretsky and colleagues described several mutations associated with acquired resistance to PD-1 blockade in melanoma [8]. In this study, the authors examined 72 patients with metastatic melanoma treated with PD-1 blockade (Pembrolizumab) and observed an initial objective response rate of 53%. Acquired resistance was observed in 15 patients (35%), as demonstrated by disease progression after an initial objective response that lasted at least 6 months. Longitudinal tumor biopsies (pre-treatment and progression) were available in four patients, and these were deeply queried via whole exome sequencing of tumor tissue or early passage cell lines and via immune profiling to gain insight into putative mechanisms of therapeutic resistance.

In these studies, the authors observed broad comparability of the overall mutational load and chromosomal loss-of-heterozygosity events in the setting of acquired resistance to anti-PD-1 based therapy, with less than 8% of non-synonymous mutations unique to progressing tumors - even in the setting of new metastatic lesions. Of note, they identified high-level mutational loss of key genes involved in immunotherapeutic responses, involving defects in antigen presentation and in interferon signalling. In one case, they identified a β2-microglobulin frameshift deletion leading to HLA class I loss, which has previously been implicated in immunotherapy resistance [9]. In two cases, JAK mutations were found and subsequently validated in vitro to confer tumor cell resistance to IFN-γ (JAK2 mutation) or IFN-α/β/γ (JAK1 mutation) despite T cell recognition of tumor antigen. Importantly, functional loss of JAK2 was associated with reduced STAT1, STAT3, and IRF1 phosphorylation, as well as failure to upregulate TAP1, HLA class I, and PD-L1 expression. This data is highly relevant, and it is certainly plausible that over longer timeframes in vivo, such effects could also compromise T cell recognition.

In addition to genomic events, the authors identified significant alterations in anti-tumor immune responses in the setting of acquired resistance to anti-PD-1 based therapy. Namely, the site of immune activity, as defined by CD8+ T cell infiltrate and/or PD-L1 expression, was almost exclusively at the tumor invasive margin at relapse. This is important, particularly in light of data from the same group regarding the importance of assessment of the distribution of CD8+ T cells within the tumor microenviroment – demonstrating a higher density of CD8+ cell infiltrate at the tumoral invasive margin at baseline and higher intra-tumoral CD8+ T cell infiltrate early on-treatment in responders to anti-PD-1 based therapy [10]. Taken together, this suggests that acquired resistance to anti-PD-1 based therapy is associated with a reversion of the tumor to a lymphocyte-excluded state. Though defects in interferon signalling were identified in this study, the link between this and the apparent T cell exclusion at time of progression was not defined. However others have described a critical role for IFN signalling in the generation of an inflamed tumor microenvironment and recruitment of leukocytes [11], thus providing the rationale to suggest that the two may be tightly related. Recent extension of this work also implicates similar IFN-response compromise caused by JAK1/2 mutations in cases of intrinsic resistance to PD-1 blockade, however in the studied cohort of melanoma and mismatch repair deficient colon cancer patients, the prevalence of such mutations was quite low (1/23 melanoma, 1/16 colon cancer) [12]. Wider surveillance is required to quantify the broader applicability of such resistance mechanisms to innate and adaptive checkpoint inhibitor resistance.

The results presented in the manuscript are provocative, though some limitations clearly exist. Though the overall study cohort was relatively large, the number of longitudinal samples available for genomic and immune analyses was quite limited – thus it is difficult to draw strong conclusions before these results are validated in larger cohorts. This problem is not unique to this study, and highlights a critical need for the global oncology community to embrace the concept of obtaining tumor samples at several time points during therapy (ideally at pre-treatment and progression, with consideration of an early on-treatment biopsy) to better understand mechanisms of therapeutic resistance [13]. Another limitation within this study was that one patient’s pre-treatment biopsy sample was obtained several months before initiating anti-PD-1 therapy, combined with interval treatment with a BRAF inhibitor – thus the observed genomic events may have been related to selective pressure from prior therapy. Nonetheless, the identification of mutational resistance events in 3 of 4 evaluable patients is strikingly reminiscent of the resistance mechanisms observed in patients on molecularly targeted agents, thus warranting very close prospective assessment for the emergence of similar or even cross-reactive resistance mechanisms in patients treated with combined modalities (ie: combination checkpoint blockade and BRAF inhibitor-based therapy). It is, however, becoming increasingly clear that a significant proportion of resistance mechanisms may not be related to genomic events. Functional reprogramming of gene expression is an increasingly-described resistance mechanism in targeted therapies [14], and there is emerging data regarding its impact on immune targets as well [6, 15]. It is also quite clear that a suite of other immunodulatory checkpoint molecules such as TIM3, LAG3, and CTLA4 may act in sequence or in concert to maintain a continued immunosuppressive state despite adequate PD-1/PD-L1 blockade [16]. Indeed, recent work from Benci et al. implicates several alternate T cell inhibitory receptors and highlights the central role for a persistent IFN/STAT1-driven network of signalling and epigenomic changes underlying PD-1-independent adaptive resistance to checkpoint blockade [17]. Analysis of larger cohorts of immunotherapy-treated patients will be required to determine the impact of such functional plasticity on adaptive resistance in this context.

This and other studies provide a strong foundation, though additional questions remain about the broader efficacy of anti-PD-1 therapy in cancer. Will tumors of lower mutational load also display prominent tumor cell-intrinsic mutational resistance mechanisms to checkpoint inhibitor therapy? To what extent does the unique microenvironment of distinct metastatic sites influence mechanisms of resistance? And what is the role of genomic and immune heterogeneity in driving differential responses to immune checkpoint blockade? Further work will be required to help answer these and other questions, and will rely heavily on longitudinal tumor sampling before and during therapy in larger cohorts of patients on immune checkpoint blockade, across cancer types. Combined research platforms exploring simultaneous molecular and spatial dynamics will help to delineate the roles played by the diverse subtypes of tumor-infiltrating leukocytes in the tumor microenvironment, some of which may have no intrinsic tumor-specificity or anti-tumor potential. As we move forward, deep profiling via genomic and immune profiling in longitudinal tumor samples should be strongly considered in discovery cohorts to help identify putative predictive biomarkers and mechanisms of resistance, with targeted profiling of top targets in larger validation cohorts. Efforts should also be made to perform parallel analyses in longitudinal “liquid biopsy” samples to identify circulating genomic and immune predictors of response. Finally, incorporation of microbiome sequencing will also be important given the increased appreciation of the gut microbiome in shaping anti-tumor immune responses.

## Abbreviations

BRAF: B-Raf proto-oncogene, serine/threonine kinase
CTLA-4: Cytotoxic T lymphocyte-associated protein 4
HLA: Human leukocyte antigen
IFN: Interferon
IRF1: Interferon regulatory factor 1
JAK: Janus kinase
PD-1: Programmed death-1
PD-L1: Programmed death-ligand 1
STAT: Signal transducer and activator of transcription
TAP1: Transporter 1, ATP binding cassette subfamily B member
